# Redox shielding unlocks damage-free photopolymerization for GelMA 3D co-cultures mimicking pancreatic tumor complexity

**DOI:** 10.1016/j.bbiosy.2026.100150

**Published:** 2026-10-11

**Authors:** Claire Lac, Petnoi Petsophonsakul, Julie Foncy, Marjorie Fanjul, Daniel Ferri-Angulo, Jeanne Aigoin, François Pattou, Yvan Martineau, Julie Kerr-Conte, Aurélie Perraud, Muriel Mathonnet, Morgan Delarue, Laurent Malaquin, Corinne Bousquet, Christine Jean

**Affiliations:** a Univ. Toulouse, CNRS, Inserm, CRCT, Toulouse, France; b LAAS-CNRS, Univ Toulouse, CNRS, Toulouse, France; c Univ. Lille, CHU Lille, Inserm U1190, EGID, Institut Pasteur de Lille, Lille, France; d Biotherapy Platform, CHU Lille, Lille, France; e EA 3842 Laboratory, Medicine and Pharmacy Faculties, Limoges University, Limoges, France

**Keywords:** GelMA 3D model, Gelatin methacryloyl, ROS-induced cell death, N-acetylcysteine, Pancreatic stellate cells, Pancreatic ductal adenocarcinoma

## Abstract

Developing physiologically relevant 3D tumor models is critical to study cell–stroma interactions driving cancer progression. In pancreatic ductal adenocarcinoma (PDAC), pancreatic stellate cells (PSCs) are key stromal actors that acquire pro-tumoral functions upon activation into cancer-associated fibroblasts (CAFs). We aimed to create a 3D co-culture model combining PSCs (or CAFs) and tumor organoids (or spheroids) that reproduces the tumor microenvironment while preserving the viability of fragile stroma cells. Gelatin methacryloyl (GelMA) was selected as a biocompatible scaffold with mechanical properties comparable to pancreatic tissue. However, fragile stromal cells, such as PSCs, displayed extensive photopolymerization-induced cell death when embedded in GelMA, preventing long-term culture. We developed a simple preconditioning protocol with an antioxidant (N-acetylcysteine, NAC) that protects sensitive cells prior to GelMA encapsulation. This approach allowed long-term 3D culture of healthy PSCs and CAFs, alone or in co-culture with tumor spheroids or organoids. Cells self-organized, maintained physiological morphology, secreted extracellular matrix, and formed stromal networks around tumor structures, closely mimicking the PDAC tissue architecture. This straightforward and reproducible approach enables sensitive stromal cells to thrive in 3D GelMA, providing a robust platform to study tumor–stroma crosstalk and to support drug testing in PDAC-relevant models.

## Highlights

• NAC preconditioning prevents PSC death and enables robust GelMA 3D culture.
• Protected PSCs/CAFs thrive and self-organize into long-lasting stromal networks.
• The model mimics PDAC architecture, with fibroblasts encapsulating tumor structures.

## Graphical abstract

A simple antioxidant preconditioning enables sensitive fibroblasts to survive GelMA encapsulation, self-organize, and form stromal networks around tumor organoids. This 3D model faithfully recapitulates pancreatic ductal adenocarcinoma microenvironment, providing a robust platform to study tumor–stroma interactions and test therapeutics.

**Figure:**
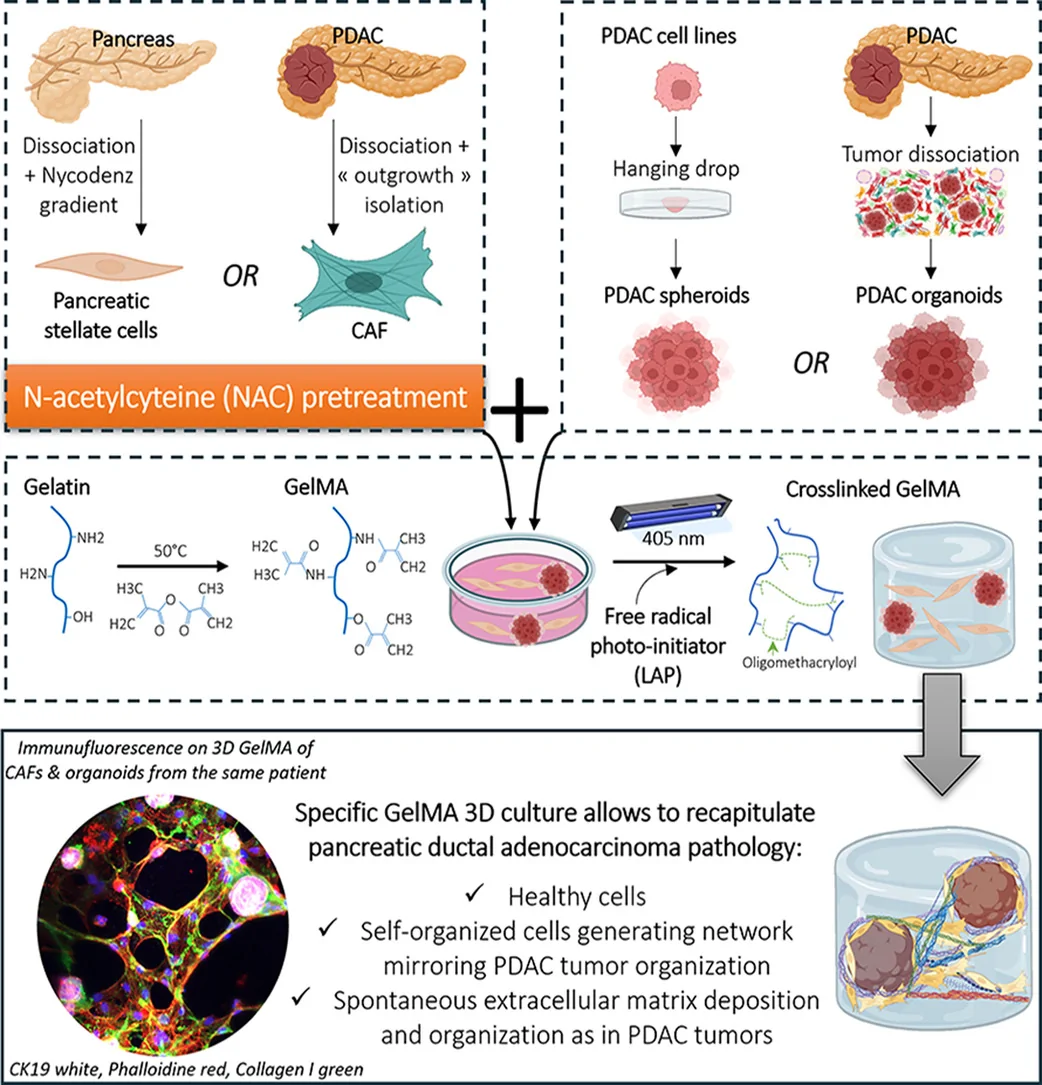

## Introduction

Significant progress has been made in the quest to develop innovative biocompatible compounds and *in vitro* models that mimic pathologies as closely as possible. While traditional research has long relied on animal models, ethical considerations and the challenge of translating results to humans have led to a reduction in their use [1]. Recognizing the limitations of traditional two-dimensional cell cultures, there is a growing need for more sophisticated three-dimensional (3D) models to deepen our understanding of complex biological processes [2].

The development of these 3D systems requires biocompatible materials, for which a broad range of natural and synthetic options are available. The use of animal-based materials such as collagen I or Matrigel® has limitations such as being expensive, inconsistent in composition, and requiring specific handling. In contrast, hydrogels, characterized by their three-dimensional cross-linked network structure, biocompatibility and biodegradability, have emerged as pivotal tools. Their porous nature and high water content allow them to mimic the extracellular matrix (ECM), facilitating cell adhesion and influencing many biological processes [3]. Hydrogels can be produced from natural polymers like polysaccharides (e.g., cellulose, alginate) and proteins (e.g., gelatin, collagen), or synthetic polymers such as polyethylene glycol (PEG), using either physical (hydrogen bonding, hydrophobic interaction, etc.) or chemical (free radical polymerization, click chemistry, etc.) cross-linking methods [4]. While certain hydrogels such as alginate, cellulose or PEG have limited binding and cell interaction capabilities and require modification [4], gelatin methacryloyl (GelMA) stands out as a state-of-the-art semi-synthetic hydrogel with remarkable processing capabilities. Its exceptional biocompatibility and degradability have positioned GelMA as a pioneering material in the advancement of tissue engineering. Originally developed in 2000 by Dr. Van de Bulcke's team, GelMA is synthetized by adding methacrylate-based groups to gelatin through chemical reactions involving amine (-NH2) and hydroxyl (-OH) groups [5]. This biomaterial contains RGD sequences (Arg-Gly-Asp peptides) that enable cell adhesion, spreading, and differentiation, as well as matrix metalloproteinase (MMP) sequences that aid in enzymatic dissociation [6]. These features ensure excellent compatibility with cells while enabling biodegradation. In addition, the methacryloyl groups allow GelMA to rapidly crosslink upon exposure to UV or visible light in the presence of a free radical–generating photoinitiator, providing fast processing, tuneable mechanical properties, design flexibility, and facile customization [6,7]. GelMA has been extensively studied and applied in fields such as bioprinting and tissue engineering [8].

Pancreatic ductal adenocarcinoma (PDAC) is one of the deadliest cancers and forms a very stiff tumor, with a Young’s modulus exceeding 10 kPa [9]. PDAC is characterized by a very dense stromal compartment (that can represent up to 80% of the tumor mass) where pancreatic stellate cells (PSCs) and cancer-associated fibroblasts (CAFs), main cellular components and major producers of extracellular matrix (ECM), play critical pro-tumoral roles [10,11]. Modelling the crosstalk between tumor cells and stromal cells requires a 3D platform that preserves cell viability while allowing organoid or spheroid integration, cell self-organization, and maintaining all of their key functions. GelMA meets these criteria, providing a matrix free of animal-derived ECM proteins yet with tissue-matched stiffness. GelMA crosslinking is typically achieved by exposing the polymer to light (405 nm, violet light at the edge of the visible spectrum) in the presence of the lithium phenyl-(2,4,6-trimethylbenzoyl) phosphinate (LAP) leading to free radical generation and crosslink of methacryloyl groups [6,7]. The important challenge we encountered is that pancreatic stromal cells, particularly PSCs isolated from human or mouse healthy pancreas, were found to be highly sensitive to the photopolymerization process, compromising their survival in GelMA.

Here we reported a simple and straightforward cell preconditioning strategy to overcome such limitations. Our approach allows stromal cells to self-organize, secrete ECM, and form networks around tumor structures, closely mimicking the PDAC microenvironment, providing a robust platform to study tumor–stroma interactions in a physiologically relevant context.

## Material and methods

### Cell culture and treatments

All cell lines were free from mycoplasma (tested using MycoAlert Mycoplasma Detection Kit, LT07–318, Lonza).

#### Human pancreatic stellate cells

Pancreatic stellate cells (PSCs) were isolated from a suspension of human dissociated pancreas. Pancreas dissociation and separation of exocrine parenchyma and Langerhans islets were performed according to the automated method of Ricordi *et al*. [12] with modifications [13] at the Biotherapy Platform, Lille Hospital, France. The PSC isolation was performed on the pancreatic suspension devoid of islets according to a slightly modified protocol [14]. Briefly, pancreas suspension was incubated during 7 min with digestion buffer (0.05% collagenase P (Sigma # 11213865001), 0.1% DNAse (Sigma # D5025), 0.02% pronase (Sigma # 10165921001) in Gey’s Balanced Salt Solution (GBSS, Sigma #G9779) freshly prepared. The digested sample was filtrated through 100 μm nylon mesh, centrifuged at 1800 rpm for 8 min and washed with 0.3% bovine serum albumin (BSA) in GBSS. After centrifugation, the pellet was resuspended in 0.3% BSA GBSS and mixed in 35,6% Nycodenz solution (Alere Technologies, # 1002424). The Nycodenz gradient was prepared by layering 22 mL of cell suspension in Nycodenz underneath 9 mL of 0.3% BSA GBSS in a 50 mL centrifuge tube. After a centrifugation at 2600 rpm for 20 min (without brake), PSCs present at the interface were harvested, washed with 40 mL of phosphate buffer saline (PBS) solution plus 1% penicillin/streptomycin (P/S, Sigma Aldrich # P0781), centrifuged 8 min 1800 rpm, resuspended in complete medium: DMEM F12 (Gibco # 31331–08) plus 10% fetal calf serum (FCS, Eurobio Scientific # CVFSVF00–01) plus 1% P/S and then seeded into 6-well plate pre-coated with poly-L-lysine (Sigma Aldrich, # P4707). Medium was changed after 45 min, and primary PSCs were expanded, frozen and/or been immortalized *via* retrovirus‐mediated expression of hTERT. PSCs were grown in adherent culture, in flasks pre-coated with 0.1% gelatin (Sigma‐Aldrich, #G2500), at 37 °C in a humified 5% CO_2_ atmosphere, in complete DMEM F12 culture medium.

#### Murine pancreatic stellate cells

Primary PSCs were isolated as previously described [14] from 8-weeks old C57BL/6J mouse pancreas (Charles River). Briefly, the pancreas from mice was harvested in PBS plus P/S (1%) and cut in small pieces in a digestion buffer (as for hPSCs) freshly prepared. Same protocol as described for human PSC isolation was used except that Nycodenz solution used was 43.75%. Mouse PSCs were expanded and maintained on dishes pre‐coated with 0.1% gelatin in complete DMEM F12 culture medium.

#### Human cancer-associated fibroblasts

CAFs were isolated from human pancreatic tumor tissues as previously described [15]. Briefly, human CAFs were isolated using explant from histologically fibrotic areas of PDAC surgically resected. Small tissue blocks were cut (0.5–1 mm^3^) using razor blade and seeded in 75 cm^2^ uncoated flask in the presence of complete DMEM F12 culture medium. Tissue explants were cultured at 37 °C in a 5% CO_2_‐air humidified atmosphere. Eighteen hours after seeding, culture medium was changed. CAFs grew out from the tissue explants 1–3 days later.

#### Pancreatic cancer cell culture and spheroid generation

Murine ductal pancreatic adenocarcinoma cell line (R211) was isolated from LSL‐K‐RasG12D/+; LSL‐p53R172H/+; Pdx1‐Cre (also referred as KPC) mice [15,16] generously provided by Dr. D. Saur (University Hospital Klinikum Rechts der Isar, Technische Universität München, Germany). Cells were maintained in DMEM high glucose (Sigma #D6429) plus 10% FCS and 1% P/S. To create the spheroids (hanging drop technics [17]), tumor cells (R211) were collected, enumerated, and centrifuged to form a pellet, which was then resuspended in DMEM high glucose / 10% FCS culture medium. Next, a 20 μL droplet of the cell suspension (1500 cells) was placed on the underside of a Petri dish lid, which was inverted onto the bottom chamber filled with PBS and left to incubate at 37 °C / 5% CO_2_ for 24 or 48 h. Once formed, spheroids were collected and incorporated into the GelMA mixture.

##### PDAC organoids

*- Isolation*: For tissue dissociation, tumor tissues from patients were washed with DMEM F12 supplemented with 1% P/S and minced into 1–2 mm^3^ pieces. Tumor fragments were then incubated with 1 mg/mL Collagenase/Dispase (Roche Diagnostics #10269638001) at 37 °C for 30 min. Enzymatic digestion was stopped by addition of cold DMEM F12 medium. Dissociated tissues were spun down (100 g, 5 min) and resuspended in Accutase (Sigma Aldrich #A6964) followed by incubation at 37 °C for 40 min. Enzymatic digestion was stopped by addition of cold DMEM F12 medium. Dissociated tissues were then passed through a 100 µm cell strainer (Corning). Dissociated cells were spun down (100 g, 5 min), resuspended in complete organoid culture medium: DMEM F12 medium with 0.05 µg/mL mEGF (Gibco #PMG8041), 0.1 µg/mL FGF-10 (Peprotech #100–26), 0.01 µM Gastrin I (Bio-TechneTocris #3006), 0.5 µM A83–01 (Bio-TechneTocris #2939), 0.1 µg/mL mNoggin (Peprotech #250–38), 1.25 mM N-acetylcysteine (Sigma Aldrich #9165), 1 µg/mL R-Spondin I (Peprotech #120–38), 10 mM Nicotinamide (Sigma Aldrich #N0636), 2% B27 Supplement (Thermo Fischer Scientific #17504–001), Wnt3a-conditioned medium (50% v/v), 5% Growth Factor Reduced Matrigel® (Corning #356231), 10 µM ROCK inhibitor Y-27632 (Bio-TechneTocris #1293823) and 1% P/S.

*- PDAC organoid culture on Matrigel®*: Cells were plated on a Matrigel® pre-coated well of 24-well plate in organoid complete medium and incubated at 37 °C-5% CO_2_. The medium (without ROCK inhibitor Y-27632) was renewed every 3–4 days. Organoids were passaged every one to two weeks: Matrigel® was digested with 1 mg/mL collagenase/dispase (37 °C, 3 h). The collagenase/dispase reaction was stopped by adding cold DMEM F12 medium. The organoids were then centrifuged at 100 g for 5 min. The cell pellet was resuspended in Accutase and incubated at 37 °C for 40 min, and enzyme reaction was stopped by adding cold medium. After centrifugation (5 min, 100 g), cells were resuspended at 1:2 dilution in organoid complete culture medium and then plated in Matrigel® pre-coated well. Organoids used in this study harbour characteristic of the classical PDAC subtype [18].

### GelMA synthesis and utilization

#### GelMA synthesis [19]

Gelatin from porcine skin type A (Sigma-Aldrich #G6144, gel strength 90–110 g bloom, Type A, lot#SLBW0296) (20 g) was dissolved in 100 mL of sodium carbonate (Sigma-Aldrich #223530) / bicarbonate buffer solution (Sigma-Aldrich #S6014) (0.25 M, pH 9.0) under stirring at 50 °C for 2 h. Methacrylic anhydride (Sigma-Aldrich #276685) at 0.1 mL/g was added under vigorously stirring at a rate of 0.1 mL/min and left to react for 3 h at 50 °C. Then, the reaction was stopped by adding deionized water (DI) (100 mL). The resulting solution was transferred to sterilised dialysis tubes with 10 kDa of cut-off (MWCO) (Thermo Fisher) and dialysed at 40 °C for 4 days. Finally, the solutions were frozen in nitrogen liquid and lyophilised for 4 days (LyoQuest −85 Plus, Telstar). The resulting products were stored at −20 °C until further use. The degree of substitution (DS) was analysed by proton nuclear magnetic resonance (^1^H-NMR) spectroscopy (500 MHz) (Bruker).

#### GelMA hydrogel preparation

Freeze-dried GelMA was dissolved (10%w/w) in complete culture medium (either DMEM F12 with 10% FCS and 1% P/S or in complete organoid culture medium, see PDAC organoid culture in GelMA section) under stirring at 37 °C for 1 h. Photoinitiator lithium phenyl-2,4,6-trimethylbenzoylphosphinate (LAP, Sigma #900889) was added to the solution (0.1%w/w of GelMA) under stirring at 37 °C for 1 h before being mixed with cells (see following sections).

#### N-Acetyl-L-cysteine pre-treatment

A solution of N-Acetyl-L-cysteine (NAC, #A7250, Sigma Aldrich) at 1 M in water was prepared under stirring at 37 °C for 2 h. The pH was then adjusted to pH 7 using 5 N NaOH solution. Cells (PSCs or CAFs) were then treated with a 5 or 25 mM solution of NAC diluted in culture medium for a 2-hour incubation at 37 °C. Following incubation, cells were washed three times with PBS before being used for subsequent experiments.

#### Cell exposure to light and LAP in 2D cultures (without GelMA)

hPSCs and mPSCs were retrieved, enumerated, and cells were suspended in a complete medium containing (or not) LAP (0.1% by weight of GelMA, considering a 10% w/w GelMA concentration) at a cell concentration of 0.1 × 10^5^ cells/mL before being seeded into wells from 12-well plates. The plates containing the cell suspension were then subjected to light exposure (or not) (FormCure lamp, 405 nm, 5.5 mW/cm²) for 1 or 2 min. Cells were then incubated at 37 °C in a 5% CO_2_‐air humidified atmosphere. Twelve hours later, medium was replaced with fresh DMEM F12 complete medium, and cells were cultured for 7 days with medium changes every 72 h.

#### Cell embedding in GelMA hydrogel

*For cell monocultures*. PSCs, CAFs, or tumor cells were harvested centrifuged at 1200 rpm for 5 min and resuspended in GelMA solution at a concentration of 1 × 10^6^ cells/mL. For photopolymerization, 100 µL of GelMA solution containing cells were dispensed into well of a 96-well plate and exposed to the FormCure lamp emitting at 405 nm with an intensity of 5.5 mW/cm² for different time point indicated in figures (1 min: 330 mJ/cm^2^, 2 min: 660 mJ/cm^2^, 5 min: 1650 mJ/cm^2^) before being incubated at 37 °C for 5 min. The resulting hydrogel cylinder was then detached from the well border using a fine-tip and transferred into a 24-well plate prefilled with 600 µL of DMEM F12 complete culture medium.

*For PSCs plus tumor spheroid co-cultures in GelMA* (schematic Fig. 3A). Preformed spheroids were transferred into a suspension of NAC-pretreated PSCs in GelMA (ratio 1 tumor cell for 2 PSCs for a total of 0.5 × 10^6^ cells in 500 µL). For immunofluorescence experiments, 10 µL of the GelMA solution containing cells were dispensed into wells of 15-well chambered µ-slide (Ibidi, #81506). Photopolymerization was then performed (FormCure lamp, 405 nm, 5.5 mW/cm) and 30 µL of DMEM F12 complete culture medium added on top. For transmission electron microscopy, 100 µL of the GelMA solution containing cells were dispensed in well from 96-well flat-bottom plate. The GelMA was photo-crosslinked (FormCure lamp) before being incubated at 37 °C for 5 min. The GelMA cylinder containing PSC / tumor spheroid co-culture was transferred into a 24-well plate prefilled with 600 µL of DMEM F12 complete culture medium.

*For culture of PDAC organoids in GelMA*. Patient organoids initially cultured in Matrigel® (24-well plate) were recovered (Collagenase/Dispase plus Accutase) as described above. Cells (500 000) were resuspended in 600 µL of 10% GelMA/0.1% LAP dissolved in complete organoid medium and distributed into a 96-well flat-bottom plate (100 µL per well). The GelMA was photo-crosslinked, before incubation at 37 °C for 5 min and then transferred into a 24-well plate prefilled with 600 µL of organoid medium (as described above). Organoids were passaged every one to two weeks at dilution 1:2: after GelMA digestion with prewarmed 3 mg/mL Collagenase type I (Sigma #C9263; 37 °C, 15 min), followed by a centrifugation (100 g, 5 min), organoids were incubated with Accutase (37 °C, 40 min), enzymatic reaction stopped by adding 1 mL serum, and cells resuspended in 10% GelMA / 0.1% LAP dissolved in complete organoid medium. Distribution in 96 well plate, photo-crosslinking, incubation at 37 °C for 5 min and transfer into 24-well plate were then performed as described above. For cryopreservation, single cell suspension of organoids was resuspended in freezing medium (Gibco #12648–010) and stored at −80 °C, then in liquid nitrogen. Cryopreserved organoid cells were rapidly thawed (37 °C), and centrifuged. Organoid cells were embedded in GelMA as described above for long-term organoid culture.

*For CAF plus organoid co-cultures* (schematic Fig. 4B). CAFs and organoids were processed in order to obtain single cell suspension (respectively, using trypsin treatment, and collagenase/dispase plus accutase treatment as described in “PDAC organoids” section). Then, 1 × 10^6^ cells/mL (consisting of 1/3 of tumor cells from human organoids and 2/3 CAFs) were resuspended in GelMA before being dispensed in well from 15-well chambered µ-slide (Ibidi, #81506; volume per well: 10 µL) for immunofluorescence experiments. After photopolymerization using the FormCure lamp, and incubation for 5 min at 37 °C, 30 µL of complete organoid culture medium was added.

*Measure of Young’s modulus.* GelMA underwent photopolymerization within 1.5 mL Eppendorf tubes, utilizing the FormCure lamp (FormLabs). This process yielded hydrogel cylinders measuring 8 mm in diameter and 5 mm in thickness. Subsequently, Young's modulus evaluations were conducted on a compression bench (MARK-10 series 5, Force gauge model M5–2 = 10 N), employing a planar configuration. A pre-stress of 0.1 N was initially applied, followed by compression of the hydrogel within its elastic limit, ensuring no permanent deformation occurred.

### Cell viability assays

#### Live and dead cell assay

Live and dead cell staining was performed according to the manufacturer's procedure (LIVE/DEAD kit; Thermo Fisher Scientific #2539082). Briefly, 2D cell cultures or 3D GelMA cultures were washed before being incubated with a mix containing calcein-AM and propidium iodide for 15 min at room temperature (RT). Cells were then imaged using Cell Observer widefield microscope (Zeiss) or Incucyte S3 (Sartorius).

#### Annexin V / Propidium Iodide staining

Single cell suspension from 2D experiments was performed through trypsin and centrifugation at 1200 rpm for 5 min. To generate single cell suspension from 3D GelMA experiments, the hydrogel was subjected to digestion. 1 mL (for 500 µL of GelMA) of collagenase type I dissolved in HBSS buffer (Sigma #H8264) at 3 mg/mL was added to the well containing the hydrogel and left for 30 min at 37 °C. Subsequently, 1 mL of cold medium was introduced to stop collagenase activity, cell suspension was recovered and washed twice with PBS and pelleted (centrifugation 1200 rpm, 5 min). Annexin V/PI assay was performed according to the manufacturer's procedure (Annexin V/PI kit; Miltenyi, #130-092-052). Briefly, cell pellet was suspended in 1X Binding Buffer and incubated in the dark at RT for 15 min with Annexin V-FITC. Cells were then washed twice with 1X Binding buffer. Just before flow cytometry analysis, PI solution was added. Cell staining was analyzed using a flow cytometer (MACSquant VYB, Miltenyi) to identify viable, apoptotic, and dead cell populations. Data interpretation was performed using FACS FlowJo™ software (BD Biosciences).

### Transmission electron microscopy (TEM)

GelMA hydrogels containing or not mPSCs and murine tumor cell spheroids (R211) were washed three times with 0.1 M cacodylate buffer (EMS #12300) at RT, then fixed for 4 h with 2.5% glutaraldehyde (EMS #16360) in 0.1 M cacodylate buffer. Samples were washed with 0.1 M cacodylate buffer, and post-fixed with 1% osmium tetroxide (Electron Microscopy Sciences #19190) in 0.1 M cacodylate buffer for 45 min, at 4 °C, then dehydrated in graded ethanol and propylene oxide and embedded in Araldite 502/Embed-812 resin (EMS #13940). Ultra-thin sections were stained with uranyl acetate and Reynold’s lead citrate and examined in a Hitachi HT7700 transmission electron microscope at 80 keV with acquisition performed on a CCD AMTXR41 camera (CMEAB, Toulouse, France).

### Immunofluorescence

Immunofluorescence analyses were conducted on PSCs alone, organoid alone or co-cultures (PSCs with spheroids or CAFs with organoids).

*hPSCs or hCAFs cultured for 3 days* (on gelatin or in GelMA) in 15 well 3D µ-slide were washed with HBSS, and cells were fixed with 10% formalin (Sigma Aldrich, #HT501128) for 10 min and permeabilized with 0.05% Triton in HBSS (Sigma Aldrich, #T9284) for 10 min at RT. After blocking with 3% BSA in HBSS (1 hour, RT), cells were incubated (overnight, 4 °C) with primary antibodies: mouse anti-alpha Smooth Muscle Actin (α-SMA, Abcam, #ab7817, 1:100) and rabbit anti-collagen I and III (Abcam, #ab34710, 1:100). After three HBSS washes, cells were incubated with secondary antibodies (goat anti-Mouse IgG or goat anti-Rabbit IgG Alexa Fluor™ 488) diluted at 1:1000 in HBSS-BSA 3% in presence of TRITC-conjugated phalloidin (Sigma Aldrich, #P1951, 1:1000 in HBSS, 45 min, RT). Following three washes with HBSS, cells were incubated with DAPI (1:1000 in HBSS, 15 min). A drop of Dako fluorescence mounting medium (#S3023) was then added in each well and images acquired using a confocal Zeiss LSM 780 microscope.

*For PSC monocultures in GelMA for 7 days*, the hydrogel cylinders were fixed using sodium periodate (Sigma Aldrich, #311448), l-Lysine (Sigma Aldrich, #W384712) and paraformaldehyde 4% solution (Sigma Aldrich, #6148) buffer for 2 h at room temperature under gentle agitation, followed by three washes with HBSS containing 0.01% Sodium azide (Sigma Aldrich, #26628-22–8). The hydrogels were subsequently embedded in low-gelling agarose (Sigma Aldrich, #50080E) and sectioned into 300 µm thick slices using a vibratome (Leica VT1200). Slices were incubated for 1 hour with TRITC-phalloidin (Sigma Aldrich, 1:1000) and DAPI (Sigma Aldrich, 1:1000) diluted in HBSS. Finally, after three HBSS washes, a drop of Dako fluorescence mounting medium (#S3023) was added and images taken using confocal LSM780 (Zeiss).

*Monocultures (hPSCs, hCAFs, organoids) or co-cultures (mPSCs plus spheroids, or hCAFs plus organoids)* were washed twice with HBSS, fixed with 10% formalin (Sigma Aldrich, #HT501128) for 10 min (for 2D cultures) or 40 min (for 3D cultures), permeabilized with 0.5% Triton in HBSS (Sigma Aldrich, #T9284) for 5 min (for 2D cultures) or 40 min (for 3D cultures) at RT. After blocking with 3% BSA in HBSS (1 hour, RT), cells were incubated (overnight, 4 °C) with primary antibodies: mouse anti-α-SMA (Abcam, #ab7817, 1:100), mouse anti- vimentin (Abcam, #ab8069, 1:100), rabbit anti-collagen I (Thermo Fisher Scientific, #PA1–29204, 1:100), and rabbit anti-collagen IV (Abcam, #ab6586, 1:100). After three HBSS washes, cells were incubated with secondary antibodies (goat anti-Mouse IgG Alexa Fluor™ 488 or goat anti-Rabbit IgG Alexa Fluor™ 488) diluted at 1:1000 in HBSS-BSA 3% in the presence or not of TRITC-phalloidin (1:1000 in HBSS, 45 min, RT). Following three washes with HBSS, cells were incubated with either rabbit anti-cytokeratin 19-conjugated Alexa Fluor® 647 (Abcam #ab192980, 1:100) or pan-cytokeratin-conjugated Alexa Fluor® 647 (Novus biologicals # NBP2–76425AF647, 1:50) for 2 h at RT. After washes, samples were incubated with DAPI (1:1000 in HBSS, 15 min). A drop of Dako fluorescence mounting medium was then added in each well and images acquired using a confocal Zeiss LSM 780 microscope.

### RT–qPCR

hPSCs were cultivated on gelatin-coated plates (0.1%), or in GelMA (10%). Cells cultivated on gelatin were either non treated or treated with TGFβ1 8 ng/mL or IL1β 1 ng/mL for 24 h and collected directly in TRIzol‐LS (Thermo Fisher Scientific, #10296010). NAC pre-treated hPSCs were cultivated in GelMA for 24 h and collected by digesting with collagenase (3 mg/mL, 30 min, 37 °C), then centrifuged and resuspensed in TRIzol‐LS. hPSC mRNA concentrations were measured using a NanoDrop (ND‐1000 from Thermo Fisher Scientific). Reverse transcription was performed with RevertAid H Minus Reverse Transcriptase (Invitrogen, # EP0451) using random hexamer primers (Invitrogen, # S0142) according to the manufacturer's instruction, qPCR was carried out on a StepOne Plus (Applied Biosystems, Life Technologies) using Owi Green FAST qPCR Premix (Ozyme, # OZYA008–5XL) and primer concentration at 0.5 μM. Primers specific to human mRNAs were purchased from Integrated DNA Technologies (IDT) and are listed on the following table:

**Table:** 

	Primer forward	Primer reverse
ACTA2	5′ –ATGACTCAAATTATGTTTGAGACTTTCA – 3′	5′ – CCAGTTGTGCGTCCAGAG – 3′
CTGF	5′ – CTGCCCTCGCGGCTTAC – 3′	5′ – CATCCCACAGGTCTTGGAACAG – 3′
IL6	5′ – TCAATGAGGAGACTTGCCTG – 3′	5′ – GCTTGTTCCTCACTACTCTCAA – 3′
LIF	5′ – ATACGCCACCCATGTCACAA – 3′	5′ – CCCTGGGCTGTGTAATAGAGAA – 3′
RPS6	5′ – CAAGAGAATGAAGGAGGCTAAGG – 3′	5′ – CTGACTGGATTCAGACTTAGAAGTAG – 3′

### Colorations and immunohistochemistry

Paraffin‐embedded tissue sections were incubated 30 min at 60 °C before being deparaffinized and rehydrated. For Haematoxylin-Eosin-Saffron (HES) staining, slides were then incubated 3 min in haematoxylin, washed 5 min in tap water, incubated 3 min in eosin, rinsed in distilled water for a few seconds. Subsequently, slides were immersed in 96% ethanol and incubated in the saffron alcohol solution (pre- warmed to 35–40 °C for 10 min) for 15 min; then dehydrated in 100% ethanol before being cleared in xylene for 5 min and mounted using Eukitt. Immunohistochemistry staining was performed using a routine automated platform (Ventana Benchmark Ultra, Roche diagnostic, Tucson, AZ, USA) using PanCK (Zytomed # MSK098, 1/200) and α-SMA (Abcam #ab7817, 1/5000) primary antibodies, and imaged using Panasonic 250 scanner (Imag'IN core Institut Universitaire Cancer Toulouse, France).

## Results

### PDAC stromal cultures fail to withstand 3D culture in GelMA: Key role of the LAP and light combination

Gelatin methacryloyl (GelMA) is a versatile hydrogel widely used for cell culture owing to its favourable biological properties, including hydrophilicity and the presence of integrin-binding motifs. It provides a well-defined and tuneable alternative to animal-derived matrices such as collagen I and Matrigel®, particularly advantageous for fibroblasts, which are prone to activation upon direct contact with native ECM proteins. GelMA crosslinking is achieved by exposing the pre-dissolved polymer in PBS or culture medium to violet visible light (405 nm) in the presence of the photoinitiator (LAP) (lithium phenyl-(2,4,6-trimethylbenzoyl) phosphinate). Upon illumination, LAP generates free radicals that initiate the crosslinking of methacryloyl groups, leading to hydrogel photopolymerization (Figure S1).

We first generated 3D GelMA samples in presence of cells, starting by adding the most sensitive and fragile cells, the pancreatic stellate cells (PSCs). We then analysed their morphology and viability. Based on our own GelMA experience, we choose to work with a GelMA and LAP concentration of 10% w/w and 0.1% w/w respectively, and a light exposure of 660 mJ/cm^2^. By day 7, PSCs exhibited an abnormal, rounded shape rather than the typical star-shaped fibroblast morphology (Fig. 1A), and live/dead staining revealed extensive cell death (Fig. 1B). Given that the mechanical properties of hydrogels can affect cell survival, we assessed whether the 3D GelMA (10% w/w GelMA, 0.1% w/w LAP) hydrogel generated using different light exposure durations possessed features compatible with cell culture. Fig. 1C showed that the GelMA Young's modulus increased as a function of light dose, and was comprised between 10 and 20 kPa (for 110 mJ/cm^2^ and 1650 mJ/cm^2^ respectively), closely matching the Young’s modulus of pancreatic tumor tissue reported to be above 10 kPa. Thus, GelMA stiffness appears to be compatible with 3D pancreatic cell culture. In parallel, the structural organization of the hydrogel was examined by TEM to evaluate its porosity, an important factor for nutrient diffusion and cell survival. 10% GelMA exhibited a structure densification as a function of light dose (Fig. 1D) with pore diameter going from 150 to 30 nm, and wall thickness from 90 to 20 nm for 330 mJ/cm^2^ and 1650 mJ/cm^2^ respectively (Fig. 1E). Although 10% GelMA exhibited a relatively compact structure with limited pore size, nutrient diffusion and cell viability are not expected to be compromised. This is due to the degradation sites within the GelMA, which are susceptible to matrix metalloproteinases (MMPs) produced by PSCs and cancer associated fibroblasts (CAFs), enabling localized matrix remodeling and degradation by encapsulated cells.

**Fig. 1:**
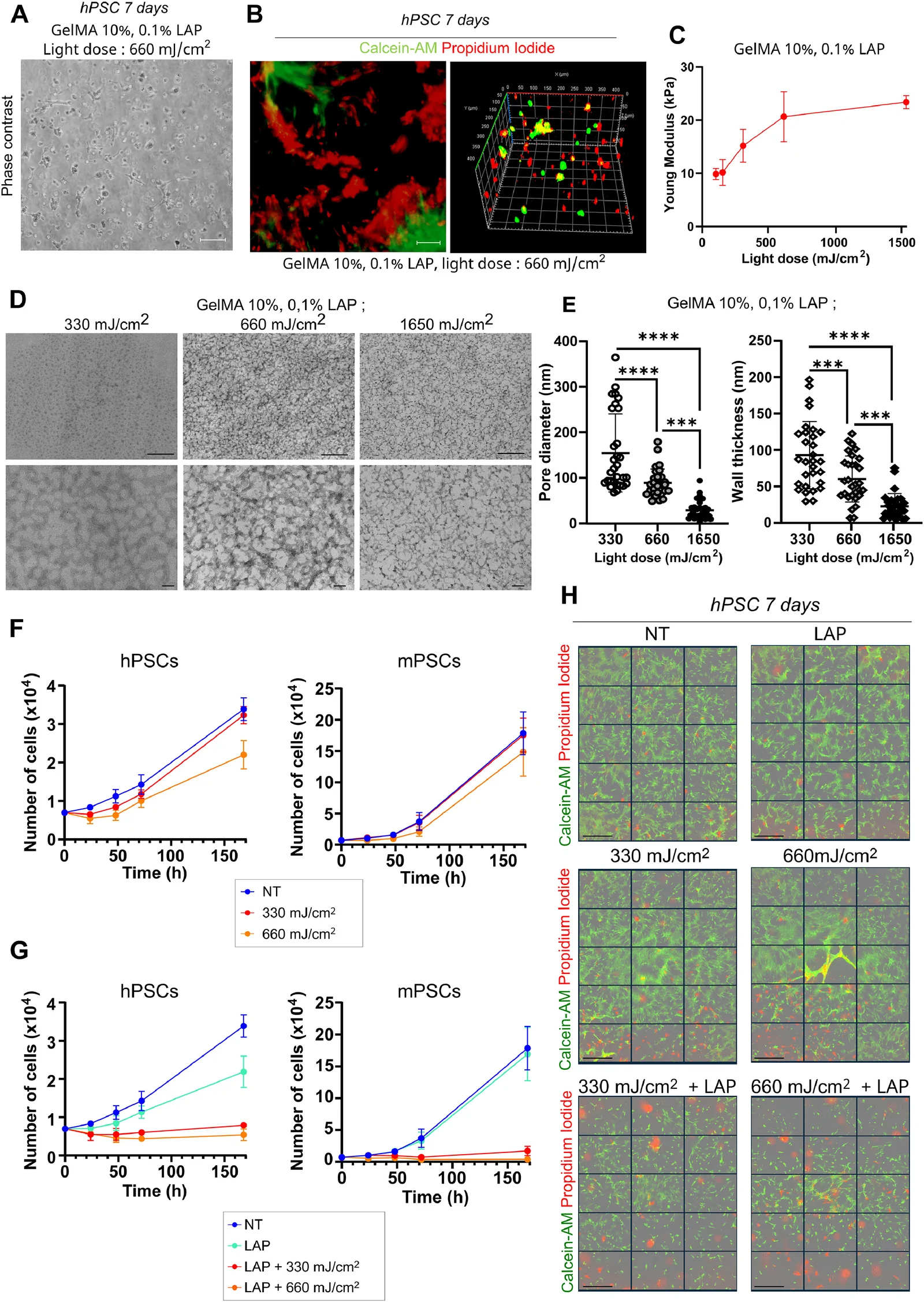
LAP and violet light combination decreases PSCs viability and proliferation.

(A) Phase contrast image of hPSCs cultured in GelMA 10% w/w (0,1% w/w LAP, 660 mJ/cm^2^ light photopolymerization) for 7 days. Image is representative of at least three independent experiments. Scale bar: 50 μm. (B) Fluorescent images (left) and 3D projection (right) of Live and Dead staining of hPSCs cultured in GelMA 10% w/w (0,1% w/w LAP; 660 mJ/cm^2^ light exposure) during 7 days. Viable cells are in green (calcein-AM staining), while dead cells are stained in red (propidium iodide). Images are representative of at least three independent experiments. Scale bar in left: 50 μm. (C) Quantification of the 10% GelMA Young's modulus as a function of light dose (mJ/cm^2^). Values are presented as means ± SD, with N = 3 independent experiments (D) Representative images of transmission electron microscopy (TEM) performed on hydrogel composed of 10% GelMA and polymerized using 0,1% LAP with different light exposure duration (from 330 to 1650 mJ/cm^2^). N= at least 3 independent wells. Scale bars on top images = 400 nm; on bottom images: 100 nm. (E) Quantification of the pore diameters and wall thickness using ImageJ. Values are means ± SD from 3 different wells. ***P < 0.001 ****P < 0.0001, by one‐way ANOVA with Tukey's method for multiple comparison. (F) Cell proliferation (cell counting on Malassez chamber) of non-treated (NT), or light-exposed hPSCs (left) and mPSCs (right) at 24 h, 48 h, 72 h and 7 days. Values are means ± SD from three independent experiments of triplicates. (G) Cell proliferation over time of hPSCs and mPSCs either non-treated, treated with LAP (0.1%), or with a combination of LAP and light (330 or 660 mJ/cm^2^). Values are means ± SD of triplicates from three independent experiments. (H) Representative image of Live and Dead staining on non-treated, light-treated, LAP-treated, or LAP/ light-treated hPSCs after 7 days of culture. Green cells are alive (calcein-AM staining) while dead cells are red (propidium iodide). Images are representative of at least three independent experiments. Scale bar: 1 mm.

As GelMA mechanical properties and structure appear to be compatible with 3D pancreatic cell cultures, we questioned the potential impact of the photopolymerization process, specifically light exposure or free radical generation by the photoinitiator LAP, on cell viability. To do so, a series of control experiments was conducted in the absence of GelMA (Fig. 1F-H). Human (hPSCs) and murine (mPSCs) PSCs resuspended in complete medium, were exposed to light at doses of either 330 or 660 mJ/cm², in the presence or absence of LAP. These conditions were designed to replicate those used during GelMA photopolymerization. Given that LAP was used at 0.1% of the GelMA mass (100 µg LAP per 100 mg GelMA in 1 mL final volume), the same concentration (100 µg/mL) was applied to cells in 2D culture.

As shown in Fig. 1F, light exposure alone had minimal impact on murine and human PSC proliferation over a 7-day period compared with non-irradiated controls. Similarly, LAP alone did not significantly alter cell growth (Fig. 1G). In contrast, the combination of LAP and light (even at the lower dose of 330 mJ/cm²) completely abrogated proliferation of both hPSCs and mPSCs (Fig. 1G). Consistent with these findings, live/dead staining revealed that cells treated with either light or LAP alone remained viable up to 7 days post-treatment, whereas those exposed to both showed extensive cell death, as evidenced by loss of calcein-AM staining and increased propidium iodide (PI) uptake (Fig. 1H).

### N- acetylcysteine pre-treatment protects PSCs and enables their 3D culture in GelMA

Our results show that the combination of light and LAP induces extensive cell death, suggesting, based on previous reports, the generation and involvement of reactive oxygen species (ROS) in this cytotoxic process. We hypothesized that inhibiting the intracellular effects of ROS could restore cell viability. N-acetylcysteine (NAC), a well-established antioxidant and cytoprotective agent against electrophiles, oxidants, and metals, neutralizes ROS either directly through scavenging or indirectly by promoting intracellular glutathione (GSH) synthesis.

To assess its potential protective effect, PSCs were pre-treated for 2 h with NAC prior to exposure to light (660 mJ/cm², i.e., 2 min) and LAP (0.1%w/w) in the absence of GelMA. NAC pre-treatment alone (at either 5 or 25 mM) had no significant impact on hPSC proliferation (Fig. 2A). Notably, while the combination of LAP and light completely abolished cell proliferation, NAC pre-treatment restored it. This recovery was more pronounced at 5 mM than at 25 mM, likely because the higher concentration induced mild cytotoxicity when combined with LAP/light treatment (Fig. 2A). Flow cytometry analysis using Annexin V/PI staining confirmed a significant improvement in cell viability following NAC pre-treatment (72% *vs*. 54% for untreated PSCs) 7 days post-exposure (Fig. 2B). These findings were further supported by Live/Dead assays performed on hPSCs after 7 days of culture (Fig. 2C).

**Fig. 2:**
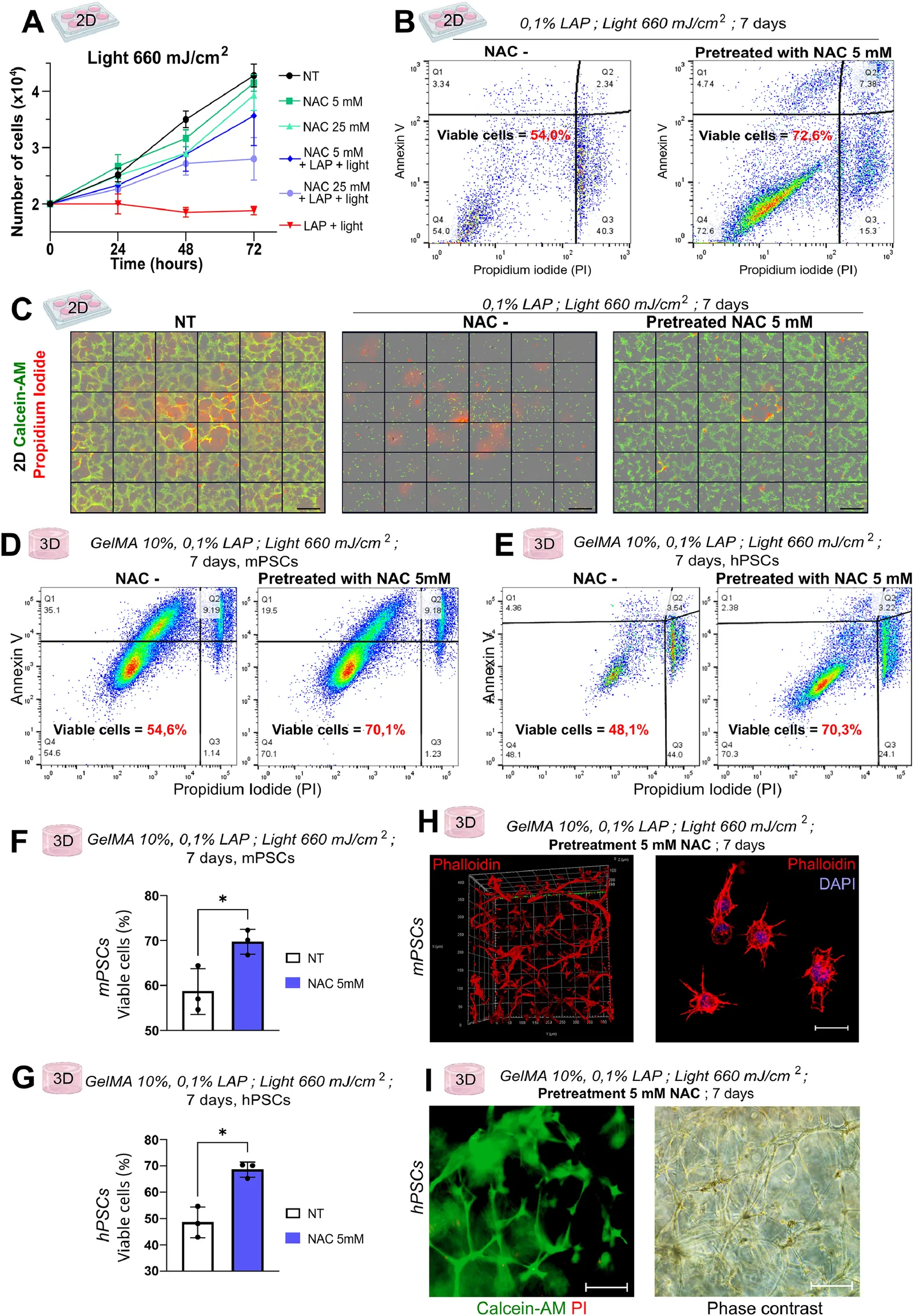
NAC pre-treatment protects PSCs and allows their culture in 3D GelMA.

(A) Impact of NAC pre-treatment on hPSC proliferation over time. Cells were pretreated or not (NT) with 5 mM or 25 mM NAC for 2 h before been subjected or not to the photopolymerization protocol (0.1% LAP; 660 mJ/cm^2^ light exposure) in well plate (without GelMA). Proliferation was evaluated through cell counting on Malassez chamber at different time point. Values are means ± SD of triplicates from three independent experiments. (B) Flow cytometry profiles of cell viability obtained after Annexin V and PI staining of hPSCs after 7 days of culture. Cells were pre-treated or not (NAC-) with NAC (5 mM, 2 h), subjected or not to the photopolymerization protocol (0.1% LAP; 660 mJ/cm^2^ light exposure) and cultured in 12-well plate (without GelMA). Graphs are representative of three biological replicates. (C) Representative images (Incucyte) of Live and Dead staining performed on hPSC after 7 days of culture. Cells were either non treated (NT), pretreated or not (NAC-) with NAC (5 mM, 2 h), and subjected to the photopolymerization protocol. Viable cells appear in green (calcein-AM staining), while dead cells in red (propidium iodide). Images are representative of at least three biological replicates. Scale bar: 1 mm. (D-E) Flow cytometry profiles of cell viability obtained after Annexin V and PI staining of mPSCs (D) or hPSCs (E) pre-treated or not with NAC (5 mM, 2 h), subjected to photopolymerisation protocol (0.1% LAP; 660 mJ/cm2 light) in presence of 10% GelMA, and cultured for 7 days in 3D. Graphics are representative of three independent experiments. (F-G) Percentage of viable mPSCs (F) or hPSCs (G) quantified based on three independent AnnexinV-PI assays performed as in D or E respectively. Values are means ± SD from three independent experiments of triplicates. *p ≤ 0.05 by independent samples *t*-test. (H) Representative immunofluorescence images of phalloidin (F-actin, red) and DAPI (nuclei, blue) performed on mPSCs pretreated with NAC (5 mM, 2 h) and embedded for 7 days in 10% GelMA hydrogel; staining was performed on thick slices of GelMA containing PSCs. Images are representative of at least three independent experiments. Scale bar in right: 20 μm. (I) Left: Representative Live and Dead staining on NAC-pretreated hPSCs cultured in GelMA for 7 days. Viable cells are depicted in green (calcein-AM staining), while dead cells are stained in red (propidium iodide). Scale bar: 100 μm.; right: Representative phase contrast images of hPSCs pretreated with 5 mM NAC and cultured in GelMA hydrogel for 7 days. Images are representative of at least three independent experiments. Scale bar: 100 μm.

We next evaluated whether NAC could confer similar protection in 3D GelMA hydrogels. hPSCs and mPSCs were pre-treated with NAC for 2 h prior to incorporation in GelMA and cultured for seven days. At day 7, hydrogels were dissociated and cell viability was analyzed by Annexin V/PI assay. Our results show that NAC provides a significant cytoprotective effect in both murine (Fig. 2D and F) and human (Fig. 2E and G) PSCs against the free radicals generated during LAP-initiated photopolymerization. Morphological analyses further supported these findings, as NAC-pretreated PSCs displayed a healthy, star-shaped fibroblast-like morphology with actin-rich protrusions and formed interconnected cellular networks within the GelMA matrix (Fig. 2H and I). Live/Dead staining confirmed these observations, revealing high cell viability and well-preserved morphology in NAC-treated samples (Fig. 2I). We also assessed the effects of photocrosslinking and NAC treatment on the activation status of the cells. As shown in Figure S2A, the mRNA expression of ACTA2 (α-SMA) and CTGF, two of the main myofibroblastic markers, was comparable in hPSCs embedded in GelMA and in 2D cultures (on 0.1% gelatin), whereas TGF-β treatment, used as a positive control, induced the expected upregulation of these markers. Similarly, the expression of the inflammatory markers IL6 and LIF was comparable between GelMA and 2D conditions (Figure S2B), whereas IL1β treatment, used as a positive control, significantly increased their expression. Immunofluorescence also confirmed that GelMA embedding did not affect α-SMA or collagen I and III expression at the protein level, in either hPSCs or hCAFs (Figures S2C-D).

Overall, these results demonstrate that NAC pretreatment effectively preserves PSC viability during photocrosslinking, thereby enabling their successful encapsulation and culture in GelMA without inducing activation or phenotypic changes.

### Pancreatic fibroblasts co-cultured with pancreatic tumor cell spheroids or organoids in GelMA recapitulate the pathology

Building on these promising findings, we sought to further complexify our model by incorporating tumor cells, either as spheroids or organoids. Since tumor cells are known to exhibit lower sensitivity to ROS, they were not pre-treated with NAC. As illustrated in Fig. 3A, pre-formed mouse tumor cell spheroids (generated using the hanging drop technique) were resuspended in medium containing GelMA, LAP, and NAC-pre-treated mPSCs (2 h, 5 mM NAC), before exposure to light (660 mJ/cm², i.e., 2 min).

**Fig. 3:**
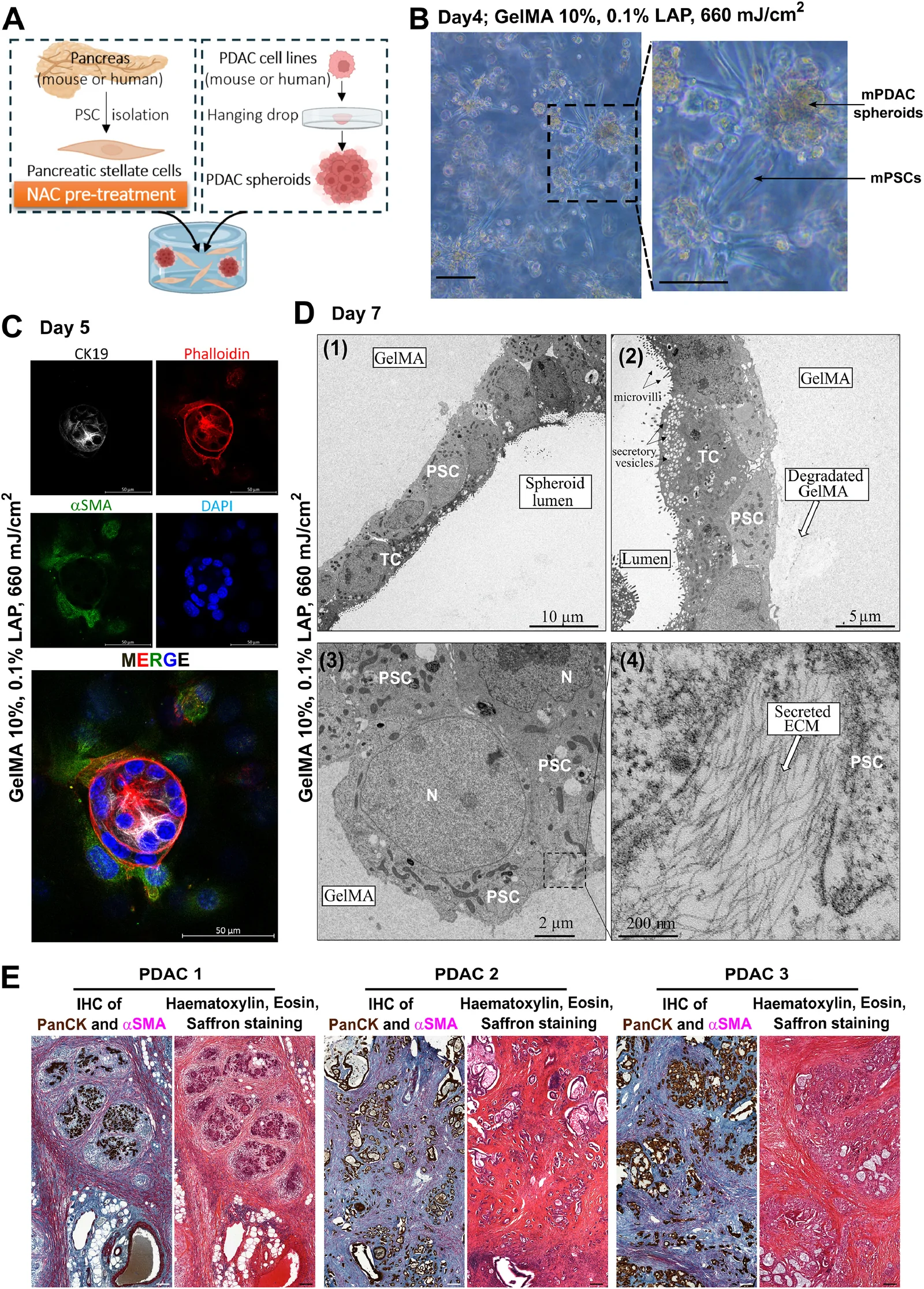
Co-culture of fibroblasts with tumor cell spheroids mimics the pathology.

(A) Schematic of the experiment: PSCs pretreated with NAC 5 mM for 2 h were embedded as single cells in GelMA in presence of pre-formed spheroids (hanging drop technic) from PDAC tumor cells. (B) Representative phase contrast picture of NAC-pretreated mPSCs co-cultured with tumor spheroids for 4 days in GelMA. Images are representative of at least three independent experiments. Right: higher magnification of left picture. Scale bars: 100 μm; (C) Representative immunofluorescence performed on NAC-pretreated mPSCs co-cultured with mouse PDAC spheroids in GelMA for 5 days. Tumor cells are identified based on the cytokeratine 19 marker (white), mPSCs based on αSMA staining (green), nuclei stained with DAPI (blue), and cell shape shown by phalloidin staining (red). Images are representative of at least three independent experiments. (D) Representative transmission electron microscopy images of NAC-pretreated mPSC co-cultured with murine tumor spheroids during 7 days in GelMA. Images are representative of at least three independent experiments. PSC: pancreatic stellate cells, TC: tumor cells, ECM: extracellular matrix, N: nuclei. Scales are indicated on images. (E) Representative immunohistochemistry (IHC) of pan-cytokeratin (panCK, brown, tumor cells) with alpha‐smooth muscle actin (αSMA, red/pink, CAFs) and of Haematoxylin, Eosin and Saffron (HES; nuclei in purple, cytoplasm in pink; ECM fibers in red/orange) staining of 3 different human PDAC tissues (PDAC1‐3). IHC and HES are serial sections from the same tumor. Scale bars: 200 μm.

Days after encapsulation, both mPSCs and tumor spheroids displayed a healthy morphology and significant intercellular reorganization, with the two populations appearing fully interconnected despite the initial embedding of mPSCs as single cells (Fig. 3B at Day 4). Between day 4 and day 7, the spheroids undergo progressive structural reorganization, including the emergence of a lumen by day 5, while the PSCs remain well organized around the epithelial spheroid (Fig. 3C). To better characterize this multicellular network, electron microscopy analyses were performed at day 7. As shown in Fig. 3D, tumor cell spheroids exhibited a well-organized architecture, including a prominent central lumen. Tumor cells displayed high polarization and abundant apical microvilli and secretory vesicles (Fig. 3D1 and 3D2). We also noticed that GelMA was absent from the spheroid core (lumen), likely because the lumen forms progressively in 3D over time and therefore does not allow GelMA to penetrate into the organoid. We further observed that GelMA appeared degraded in areas adjacent to mPSCs (Fig. 3D1 and 3D2), suggesting that the hydrogel was digested by matrix metalloproteinases (MMPs) secreted by PSCs and/or tumor cells. Moreover, electron microscopy also revealed that PSCs co-cultured with tumor cell spheroids in GelMA have the ability to secrete ECM, recapitulating the main fundamental function of PSCs once activated by tumor cells (Fig. 3D3 and 3D4). Importantly, immunofluorescence and TEM experiments show that PSCs self-organized around the cancer cell spheroids (Fig. 3B-D), closely resembling the characteristic organization observed in PDAC, where fibroblasts form a dense capsule surrounding tumor cell cyst (Fig. 3E).

**Fig. 4:**
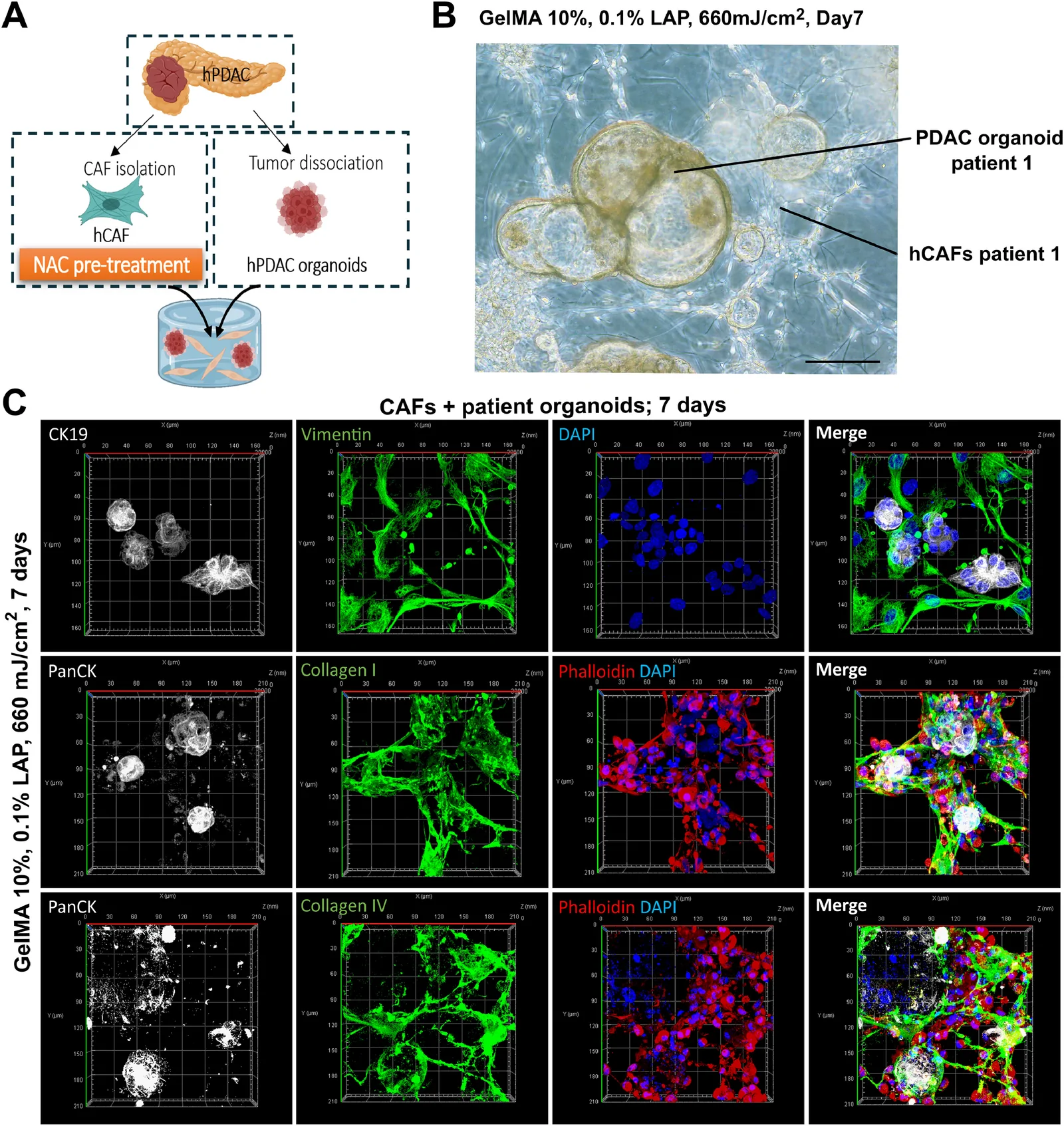
Co-culture of cancer associated fibroblasts with tumor organoids in GelMA recapitulates the pathology.

(A) Schematic of the experiments: hCAFs pretreated with NAC 5 mM for 2 h and tumor cells from human PDAC organoids were embedded as single cells in GelMA. (B) Representative phase contrast picture of NAC-pretreated hCAFs co-cultured with tumor organoids during 7 days in GelMA. Images are representative of at least three independent experiments. Scale bar: 100 μm; (C) Representative images of immunofluorescence 3D projections, generated from co-cultures of NAC-pretreated hCAFs and human tumor organoids in GelMA at 7 days of culture. Tumor cells were stained with either cytokeratin 19 (CK19) or pan-cytokeratin (PanCK) antibodies. CAFs are identified based on their vimentin expression, or very high phalloidin staining. Extracellular matrices were stained, as indicated using anti-collagen I or IV antibodies. Images are representative of at least three independent experiments. Scales are indicated on images.

To further assess the robustness of our protocol, we next co-cultured patient-derived PDAC organoids with matched CAFs isolated from the same human tumor (Fig. 4A). CAFs were pre-treated with NAC according to the established protocol, while organoids were cultured in PDAC organoid standard medium. After 7 days in GelMA (Fig. 4B), organoids and CAFs self-organized into a spatial arrangement resembling that observed in human PDAC tumors (Fig. 3E), with CAFs encapsulating tumor cysts whose central lumen is characteristic of the classical PDAC subtype used in this study. Immunofluorescence confirmed these observations, showing CAFs surrounding the organoids (Fig. 4C). CAFs were identified by vimentin or high phalloidin, while organoids were identified by cytokeratin-19 (CK19) or pan-cytokeratins (pan-CK) expression. Notably, we noticed an important deposition of collagen I and IV, mainly located around the CAF network (phalloidin^high^ cells), which is in accordance with the fact that CAFs are the main producer of ECM (Fig. 4C middle and bottom, Supplementary Videos 1 & 2). Of note, during this work we found that patient-derived PDAC organoids can be maintained and subcultured in GelMA for at least one month (Figure S3A). GelMA-embedded organoids retained morphology, organization (Figure S3A & C), and viability comparable to those observed in Matrigel® (Figure S3B & C).

Altogether, our results demonstrate that the co-culture of NAC-pretreated fibroblasts with tumor cell spheroids or organoids in GelMA successfully recapitulates key histological and functional features of the native PDAC stroma, providing a physiologically relevant 3D model to study tumor–stroma interactions.

## Discussion

Gelatin methacryloyl (GelMA) is emerging as a highly promising biomaterial to recapitulate certain key features of the extracellular matrix. Its ease of use and interesting mechanical properties enhance its appeal, particularly in fields such as oncology research. In addition, the presence of adhesion sites and matrix metalloproteinase-degradable motifs supports key cellular processes including migration, differentiation, and proliferation [[20], [21], [22], [23], [24], [25]]. A major advantage of GelMA lies in the high degree of control and reproducibility achievable during its synthesis, particularly regarding its compositional, structural, and functional properties [22]. This contrasts with materials such as Matrigel®, whose batch-to-batch variability can compromise experimental consistency. Owing to these attributes, GelMA has been successfully applied in 3D printing, injectable hydrogels for tissue regeneration, micropatterning, and electrospinning, and can be tailored to mimic tissues of varying stiffness, from bone or liver to vascular and skin tissues.

Despite these advantages, our attempt to develop a 3D co-culture model of pancreatic stellate cells (PSCs) and tumor cells revealed a major limitation: a substantial death of the PSCs after GelMA photopolymerisation. Several studies have shown that both the intensity and duration of light exposure used for GelMA polymerization could influence cell survival [[26], [27], [28]]. While low-dose irradiation has been reported to be non-cytotoxic and not to alter fibroblast or mesenchymal stem cell proliferation, apoptosis, or proteomic profiles [29,30], the formation of free radicals such as reactive oxygen species (ROS), appears to be the primary driver of toxicity [6,7,31,32]. Indeed, the generation of ROS, due to the effect of the light on the photoinitiator, is a required step for GelMA crosslinking. While tumor cells are known to exhibit high resistance to ROS [33], other cells, such as fibroblasts may be sensitive. Indeed, high concentrations of free radicals are well known to damage cellular structures, nucleic acids, lipids, and proteins [34], and several studies have reported similar cytotoxic effects associated with photopolymerization processes [29,35,36]. Strategies such as systematic washing after photopolymerization [7,30], or supplementing culture medium with ascorbic acid have been proposed to mitigate radical-induced toxicity, improving, for example, the survival of human aortic smooth muscle cells in PEGDA-based hydrogels. However, because ascorbic acid can interfere with hydrogel gelation [37], alternative approaches are required for GelMA systems.

To address this issue, we developed a protocol designed to protect cells from ROS without altering GelMA crosslinking. Our strategy relies on pre-treating cells with N-acetylcysteine (NAC), an antioxidant and free-radical scavenger that acts directly or indirectly through the elevation of glutathione intracellular levels [[38], [39], [40]]. This intracellular mechanism makes NAC particularly suitable for GelMA-based constructs, as it shouldn’t interfere with the free-radical polymerization reaction required for hydrogel stabilization [40]. Using this approach, we were able to culture PSCs and CAFs in 3D, alone or together with tumor cells, for up to 7 days while preserving viability and physiological morphology. Furthermore, PSCs and CAFs retained their capacity to self-organize around tumor aggregates and to remodel their environment by degrading GelMA (very likely through matrix metalloproteinases (MMPs) secretion [41]) and depositing their own ECM, thereby recapitulating key architectural features of pancreatic ductal adenocarcinoma (PDAC).

Building on this optimized protocol, more complex 3D co-culture models can now be envisioned to better recapitulate PDAC pathology. For instance, previous work has demonstrated the formation of microvascular capillaries in GelMA through the co-culture of HUVECs (Human Umbilical Vein Endothelial Cells) with bone-marrow-derived stem cells capable of differentiating into pericyte-like cells [42]. Yet, limited information exists regarding the impact of photopolymerization on mature endothelial cells encapsulated in GelMA, and given their well-established sensitivity to radicals, NAC pretreatment or even treatment during GelMA photopolymerization (as its action primary occurs intracellularly [40]) may prove particularly valuable for maintaining endothelial integrity during crosslinking. Moreover, increasing interest is being directed toward the role of nerves in PDAC, as the mechanisms underpinning tumor/nerve crosstalk remain insufficiently understood. GelMA represents an attractive platform for such studies, given its ability to maintain the stemness of human neural progenitors and support neuronal differentiation [20,25]. Notably, co-culturing dorsal root ganglia neurons with Schwann cells in GelMA enables prolonged interactions and enhanced myelination compared to substrates such as poly-d-lysine or collagen [43].

Altogether, the protocol we describe here opens the possibility of reconstructing, in 3D, the complex PDAC microenvironment within a microphysiological system integrating tumor cells, PSCs, CAFs, endothelial structures, and neural components.

## Funding

This work was supported by LNCC (Ligue Nationale Contre le Cancer R21030BB/RAB21012BBA), and the French National Institute of Cancer (PCSI2019 number ASC19036BSA and PLBIO23 R24012BB/RPT24002BBA). This work was also partly supported by the French National Agency for Research (Printiss Project, grant ANR21 CE1941), LAAS-CNRS micro and nanotechnologies platform, a member of the French Renatech network and by the LAAS-CNRS characterization platform. It was partly supported as part of the MultiFAB project funded by FEDER European RegionalFunds and French Région Occitanie (grant agreement number 16007407/MP0011594) and by the HoliFAB project funded by the European Union’s Horizon 2020 research and innovation program (grant agreement No. 760927).

## Ethics declaration

Written informed consent to take part in the study and to publish the article has been obtained from all participants or their legal representatives. The privacy rights of participants have been observed.

This study included organ or tissue donors. This study includes human biological material and consent was obtained from donors, or their next of kin or legal representatives, for use in this study and for publication of the article. The samples used in this research were not sourced from executed prisoners or prisoners of conscience.

This study was performed in compliance with relevant laws, regulatory frameworks and guidelines where the research took place. This study is part of a research protocol approved by the French regulations on the Lille University Hospital site on the subject of “Design, development and optimization of production and evaluation tools to improve islet transplantation and evaluation of islet-parathyroid co-transplantation” # PFS16–008. This study is also part of a research protocol approved by the Comite de protection des personnes sud-ouest et outre mer II. (Approval No. DC-2016-–2654).

## CRediT authorship contribution statement

**Claire Lac:** Writing – review & editing, Writing – original draft, Validation, Methodology, Investigation, Formal analysis, Conceptualization. **Petnoi Petsophonsakul:** Writing – review & editing, Validation, Investigation, Conceptualization. **Julie Foncy:** Writing – review & editing, Supervision, Investigation, Formal analysis, Conceptualization. **Marjorie Fanjul:** Writing – review & editing, Writing – original draft, Supervision, Investigation, Conceptualization. **Daniel Ferri-Angulo:** Writing – review & editing, Resources, Methodology. **Jeanne Aigoin:** Investigation. **François Pattou:** Resources, Methodology. **Yvan Martineau:** Methodology, Resources. **Julie Kerr-Conte:** Resources, Methodology. **Aurélie Perraud:** Writing – review & editing, Resources, Methodology. **Muriel Mathonnet:** Resources, Methodology. **Morgan Delarue:** Writing – review & editing, Writing – original draft, Supervision, Resources, Methodology, Investigation, Funding acquisition, Conceptualization. **Laurent Malaquin:** Writing – review & editing, Resources, Methodology. **Corinne Bousquet:** Writing – review & editing, Supervision, Resources, Funding acquisition, Conceptualization. **Christine Jean:** Writing – review & editing, Writing – original draft, Visualization, Validation, Supervision, Resources, Methodology, Investigation, Funding acquisition, Formal analysis, Data curation, Conceptualization.

## Declaration of competing interest

The authors declare that they have no known competing financial interests or personal relationships that could have appeared to influence the work reported in this paper.

## Data Availability

Data will be made available on request.

## Appendix Supplementary materials

Figure S1: Schematic of the generation of the GelMA hydrogel. Violet light (405 nm) was applied on the GelMA solution containing the photoinitiator LAP at a concentration of 0.1% of GelMA mass allowing the release of reactive oxygen species (ROS) by the photoinitiator LAP and the subsequent crosslinking of GelMA molecules *via* methacrylate groups.

Figure S2: (A) & (B) Quantitative real‐time PCR gene expression analysis of hPSCs cultured in different conditions: on gelatin (2D) ± TGFβ (8 ng/mL) ± IL1β (1 ng/mL), or in GelMA. Myofibroblastic (ACTA2 and CTGF in (A)) and inflammatory (IL6 and LIF in (B)) gene expressions were analyzed. The normalized fold change was calculated using the delta‐delta Ct method with RPS6 as a control gene. Values are means ± SD of triplicates from three independent experiments. (C) & (D) Representative immunofluorescence images generated from hPSC or hCAFs cultured during 3 days either in 2D (on gelatin) or in GelMA (with NAC-pre-treatment). Cells were stained with either α-SMA (C) or Collagen I and III (D) antibodies plus phalloidin and DAPI. Images are representative of three replicates. Scale bar: 100 µm.

Figure S3: (A) Representative phase contrast pictures of PDAC patient organoids grown in GelMA for more than one month period; scale bar: 100 µm. (B) Representative phase contrast pictures of PDAC patient organoids grown on Matrigel® at day 3 and day 7 after splitting; scale bar: 100 µm. (C) Representative immunofluorescence images generated from human organoids cultured either in GelMA (top) or on Matrigel® (bottom). Cells were stained with CK19 antibody (white) plus phalloidin (red) and DAPI (blue). Scale bar: 50 µm.

Supplementary Videos 1 & 2: 3D renderings of z-stack acquisitions corresponding to Figure 4C. PanCK staining in white, Collagen I (Video S1) or IV (video S2) in green, phalloidin in red and dapi in blue. Each stack consists of 35 optical sections spanning 30 µm.
